# Transcatheter Edge-to-Edge Mitral Valve Repair versus Minimally Invasive Mitral Valve Surgery: An Observational Study

**DOI:** 10.3390/jcm13051372

**Published:** 2024-02-28

**Authors:** Miriam Silaschi, Franca Cattelaens, Hossien Alirezaei, Johanna Vogelhuber, Susanne Sommer, Atsushi Sugiura, Max Schulz, Tetsu Tanaka, Mitsumasa Sudo, Sebastian Zimmer, Georg Nickenig, Marcel Weber, Farhad Bakhtiary, Nihal Wilde

**Affiliations:** 1 Department of Cardiac Surgery, Heart Center Bonn, 53127 Bonn, Germany; miriam.silaschi@ukbonn.de (M.S.); s4frcatt@uni-bonn.de (F.C.); hossien.alirezaei@ukbonn.de (H.A.); farhad.bakhtiary@ukbonn.de (F.B.); 2 Department of Internal Medicine II, Heart Center Bonn, University Hospital Bonn, Venusberg-Campus 1, 53127 Bonn, Germany; johanna.vogelhuber@ukbonn.de (J.V.); atsushi.sugiura@ukbonn.de (A.S.); max.schulz@hotmail.com (M.S.); ta.chi.tsu.tetsu@gmail.com (T.T.); m.s-sudo@fine.ocn.ne.jp (M.S.); sebastian.zimmer@ukbonn.de (S.Z.); georg.nickenig@ukbonn.de (G.N.); marcel.weber@ukbonn.de (M.W.); 3 Department of Cardiac Surgery, Bundeswehrzentralkrankenhaus Koblenz, 56072 Koblenz, Germany; skoelsch@email.de

**Keywords:** innovations, minimally invasive cardiac surgery, microinvasive cardiac surgery, edge-to-edge repair, mitral valve surgery, mitral regurgitation

## Abstract

**Background:** Minimally invasive mitral valve surgery (MIC-MVS) has been established as preferred treatment of mitral regurgitation (MR), but mitral transcatheter edge-to-edge valve repair (M-TEER) is routinely performed in patients at high surgical risk and is increasingly performed in intermediate risk patients. **Methods:** From 2010 to 2021, we performed 723 M-TEER and 123 isolated MIC-MVS procedures. We applied a sensitivity analysis by matching age, left ventricular ejection fraction (LVEF), EuroSCORE II and etiology of MR. **Results:** Baseline characteristics showed significant differences in the overall cohort (*p* < 0.01): age 78.3 years vs. 61.5 years, EuroSCORE II 5.5% vs. 1.3% and LVEF 48.4% vs. 60.4% in M-TEER vs. MIC-MVS patients. Grade of MR at discharge was moderate/severe in 24.5% (171/697) in M-TEER vs. 6.5% (8/123) in MIC-MVS (*p* < 0.01). One-year survival was 91.5% (552/723) in M-TEER vs. 97.6% (95/123) in MIC-MVS (*p* = 0.04). A matching with 49 pairs (n = 98) showed comparable survival during follow-up, but a numerically higher mean mitral valve gradient of 4.1 mmHg (95% CI: 3.6–4.6) vs. 3.4 mmHg (95% CI: 3.0–3.8) in M-TEER (*p* = 0.04). **Conclusions:** Patients undergoing M-TEER had lower one-year survival than MIC-MVS, but differences disappeared after matching. Reduction in MR was less effective in M-TEER patients and postprocedural mitral valve gradients were higher.

## 1. Introduction

Mitral regurgitation (MR) is one of the most prevalent valvular heart diseases and is associated with an increased risk of mortality and poor quality of life, irrespective of the underlying anatomy or etiology. Minimally invasive mitral valve surgery (MIC-MVS) through a right lateral thoracotomy is currently the treatment of choice for operable patients with MR and shows excellent long-term outcomes with fast recovery and equal repair rates when compared to sternotomy [1,2,3,4]. In fact, the majority of mitral valve procedures is performed through a minimally invasive access route in Germany [5]. Through this route, repair and replacement of the mitral valve are both possible and the procedure can be performed safely even in elderly patients and re-operative procedures. However, many patients with severe MR are inoperable or at increased surgical risk: accordingly, transcatheter therapies were developed to address undertreatment of this patient population. Mitral transcatheter edge-to-edge repair (M-TEER) interventions are a routinely performed alternatively to conventional mitral valve surgery in high-risk patients [6,7]. These procedures are a less invasive therapy for MR patients. Reduction in MR by use of M-TEER is associated with improved clinical outcome [8]. European guidelines recommend consideration of transcatheter edge-to-edge repair (TEER) only for high-surgical-risk or inoperable patients in both primary and secondary MR (class IIb of recommendation) [7]. American guidelines tend to favor the transcatheter procedure in patients with primary or secondary MR and depressed left ventricular function (class IIa of recommendation) [6].

Nevertheless, previous studies comparing outcome data on mitral interventions and surgery did not include a minimally invasive approach. Data comparing MIC-MVS and M-TEER are limited, no study to date has directly compared those two treatment modalities so often performed in heart valve centers across Europe. In fact, heart teams should be made aware of the advantages and disadvantages of both treatment options.

We studied the effect of MIC-MVS and M-TEER on patients with MR and compared one-year survival as well as MR reduction after interventional or minimally invasive surgical MR treatment in an elderly patient cohort.

## 2. Material and Methods

### 2.1. Study Design and Patient Cohort

This study was designed as a retrospective analysis of prospectively and consecutively collected patient data from the Heart Centre Bonn. Patients treated for MR with M-TEER or isolated minimally invasive mitral valve surgery between 2010 and 2021 were included. All patients undergoing M-TEER procedures from 2010 to 2021 were included (n = 723) and were considered as inoperable or at high risk for surgery by the interdisciplinary heart team. After a standardized diagnostic workup, including transthoracic (TTE) and transesophageal echocardiography (TEE), and left-heart and right-heart catheterization, each patient’s anatomical suitability for a catheter-based valve repair system was assessed using computed tomography (CT) images. The indication for a mitral valve intervention was evaluated based on the current guidelines, and the decision to perform transcatheter mitral valve interventions as well as the device selection was made by the interdisciplinary heart team.

In the surgical group, procedures via median sternotomy and patients with endocarditis were excluded. This yielded 123 MIC-MVS patients (Supplementary Table S1). We excluded procedures performed through median sternotomy since mitral valve surgery procedures through median sternotomy are not currently considered standard of care at the participating center, since they are known to carry a higher risk of morbidity and lead to slower postoperative recovery and mobilization [9]. MIC-MVS became standard of care in our center in 2018.

### 2.2. Procedure

Details of MIC-MVS have been well described [10]. Procedures were performed under general anesthesia; MIC-MVSs were performed in cardiac surgical operating theatres. MIC-MVS procedures were performed via the 3rd or 4th intercostal space (as suitable according to pre-operative CT) under extracorporeal circulation, which was established under full heparinization via the femoral vessels and an additional venous cannula through the jugular veins for bicaval venous drainage in case of additional tricuspid valve repair or in patients > 80 kg body weight. Femoral access was either cut-down or percutaneous, depending on the preference of the operating surgeon. Procedures were performed using 3D visualization with a camera introduced through an intercostal port. Various mitral valve repair techniques were employed depending on the patients’ individual anatomy and pathology; valves were replaced whenever considered necessary by the operating surgeon.

M-TEER procedures were performed in our dedicated hybrid operating room via the femoral vein under TEE guidance. Various techniques and devices were used for M-TEER, depending on individual patient anatomy and availability of the devices at the time: the transcatheter procedures were performed with either the MitraClip System (Abbott Structural Heart, Santa Clara, CA, USA) or PASCAL Implant System (Edwards Lifesciences, Irvine, CA, USA). The details of each device system and procedure have previously been well described [11,12].

### 2.3. Study Endpoints

All patients were followed up through interviews at scheduled hospital visits or on the telephone, or documentation from the referring general practitioners.

The primary endpoints of this study were all-cause mortality at 30 days and one year as well as MR reduction at discharge and one year.

Secondary endpoints were postoperative bleeding, stroke, renal failure, cardiac rehospitalization and re-intervention or operation on the mitral valve.

### 2.4. Ethical Consideration

Data collection, analysis and follow-up of patients after catheter-based and surgical mitral valve procedures was approved by the local ethics committee (application No. 169/20). This study was conducted in accordance with the Declaration of Helsinki and its amendments, and all patients provided written informed consent.

### 2.5. Statistical Analysis

All data were retrospectively collected and analyzed. Data are presented as absolute numbers and percentages for categorical variables with a denominator specified for each value. Continuous variables are given as mean values with 95% lower and upper confidence intervals unless stated otherwise. Dichotomous variables were compared by Fisher’s exact test and X^2^ test wherever suitable, and continuous variables by paired and unpaired *t*-test wherever suitable. *p*-values are reported without correction for multiple testing, whereas level of significance was set to 2-tailed *p* < 0.05. A case–control 1:1 matching on behalf of prognostic and anatomically relevant variables (age, left ventricular ejection fraction (LVEF), EuroSCORE II and etiology of MR (primary/secondary)) was performed to create more comparable cohorts of M-TEER and MIC-MVS for sensitivity analysis. Maximum allowable difference between variables was 4 years in age, 5%-points for LVEF and 1.5%-points in EuroSCORE II. The 1:1 matching using the MedCalc software (version 22.021) is automatic and uses a greedy matching algorithm within these specified caliper distances, which may be manually entered and chosen. There are no replacements. The etiology of MR had to be an exact match. Matching was automatically performed using the MedCalc software (MedCalc Software Ltd., Ostend, Belgium) (Supplementary Table S1). Analysis of baseline variables was then repeated to check for balance. A covariate balance check is depicted in Supplementary Figure S1.

No methods were employed to account for any possible correlation that may arise between the two groups. Kaplan–Meier estimation was used for survival analyses, and curves were compared using the log-rank test. Median follow-up was calculated using the Shemper and Smith method. All computation was carried out using the statistical softwares GraphPad Prism 5.0 (GraphPad Software Inc., La Jolla, CA, USA), SPSS 19.0 (IBM, Armonk, NY, USA) and MedCalc (MedCalc Software Ltd., Ostend, Belgium).

## 3. Results

### 3.1. Baseline Characteristics

The unmatched cohort showed higher rates of significant differences in baseline characteristics: M-TEER patients were significantly older than MIC-MVS patients, had a lower mean GFR, a higher EuroSCORE II, lower TAPSE and higher rates of cardiovascular risk factors; many other differences are depicted in Table 1. Of note, patients with mitral surgery were more often included in the early years of the analysis, while patients with TEER were more often included later. After case–control matching as a sensitivity analysis, there were 49 patients in each group.

Especially etiology of MR and baseline LVEF was different, as M-TEER patients more often had secondary MR and a lower LVEF. Left ventricular end-diastolic volume at baseline was 136.1 mL (95% CI: 130.8–141.4) in M-TEER vs. 130.6 mL (119.9–141.2) before matching (*p* = 0.356) and 113.3 (100.4–126.3) in M-TEER vs. 125.8 (110.3–141.3) in MIC-MVS after matching (*p* = 0.215). Baseline EROA was 0.33 cm^2^ (0.31–0.34) in M-TEER vs. 0.48 (0.39–0.56) in MIC-MVS before matching (*p* < 0.01) and was 0.38 (0.32–0.45) in M-TEER vs. 0.42 (0.35–0.49) in MIC-MVS after matching (*p* = 0.43).

### 3.2. Procedural Results

In M-TEER, a MitraClip device was used in the majority of patients 95.6% (691/723). Placement of two or more clips was necessary in 47.4% (343/723) in the overall cohort and 51.1% (25/49) in the matched cohort. In MIC-MVS, the mitral valve was more frequently repaired than replaced 121/123 (98.4%) vs. 2/123 (1.6%). No conversion to open surgery occurred in M-TEER and six conversions to sternotomy occurred in MIC-MVS due to bleeding (4.9%). Procedure times showed significant differences as expected: 77.5 min (95% CI: 72.8–82.1) vs. 273.1 min (95% CI: 260.4–285.7), *p* < 0.01. Procedural data is shown in Table 2.

### 3.3. Primary Endpoints

**Figure 1:**
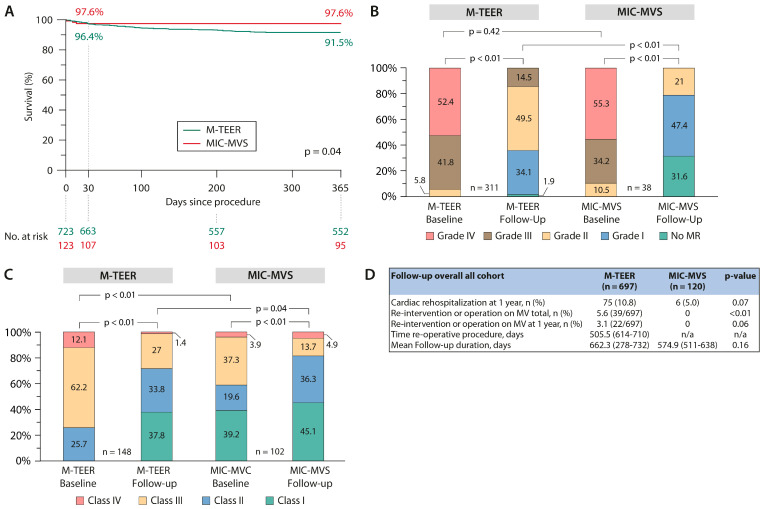
Study results of the overall cohort. (**A**) Kaplan–Meier curves of one-year survival in the mitral transcatheter edge-to-edge valve repair (M−TEER) and minimally invasive mitral valve surgery (MIC−MVS) groups. (**B**) Changes in the severity of MR grades at one year follow-up for both procedures as paired analyses. (**C**) Distribution of NYHA classes at baseline and one year as paired analyses. (**D**) Follow-up data. Legend: M-TEER = mitral transcatheter edge-to-edge repair valve repair; MIC−MVS = minimally invasive mitral valve surgery; NYHA = New York Heart Association; MR = mitral regurgitation; MV = mitral valve.

There were no significant differences in 30-day mortality, with 3.6% (26/723) in M-TEER vs. 2.4% (3/123) in MIC-TVS, (*p* = 0.78) in the unmatched cohorts. Kaplan–Meier survival analyses showed a 91.5% survival rate at one year in M-TEER and 97.6% in MIC-MVS (*p* = 0.04) (Figure 1A).

Reduction to at least moderate degree was achieved in 94.1% (656/697) in M-TEER and in 98.1% (105/107) in MIC-MVS (*p* < 0.01). At discharge, MR was reduced to none or mild in 75.3% (525/697) in the M-TEER group and in 92.5% (99/107) in the MIC-MVS group (*p* < 0.01). The mean mitral valve gradient was comparable in the overall cohort, being 3.8 (95% CI: 3.6–3.9) after M-TEER vs. 3.6 (95% CI: 3.3–3.9) after MIC-MVS, *p* = 0.32.

At follow-up, MR reduction in the overall cohort to at least moderate degree was achieved in 85.5% (266/311) in M-TEER vs. 100% (38/38) in MIC-MVS, *p* < 0.01. MR reduction to none or mild was 36.0% in M-TEER (112/311) vs. 79.0% (30/38) in MIC-MVS (*p* < 0.01) (Table 2). Follow-up for MR grading was performed at 12 months.

There was significant improvement in NYHA class during follow-up in both cohorts (Figure 1C).

Stroke occurred in 0.8% (6/723 vs. 1/123) of both M-TEER and MIC-MVS. Major bleeding events (BARC 3 or 4) were less frequently observed after M-TEER (*p* < 0.01). Three patients (0.4%) after M-TEER had low-cardiac output or need for mechanical circulatory support in the postoperative period, whereas five patients needed veno-arterial extracorporeal membrane oxygenation (VA-ECMO, 4.1%) and one patient (0.8%) needed left ventricular unloading using a microaxial pump in the MIC-MVS cohort. In-hospital stay was significantly shorter in the M-TEER cohort (Table 2).

Cardiac-related rehospitalization at one year occurred in 10.8% of M-TEER vs. 5.0% of MIC-MVS patients (*p* = 0.07). Re-intervention of operation on the mitral valve occurred in 5.6% of patients in M-TEER and in no patient in MIC-MVS (*p* < 0.01); however, re-intervention or operation rate at 12 months was 3.1% vs. 0% (*p* = 0.06) (Figure 1D).

### 3.4. Sensitivity Analysis—Case Control Matching

Additionally, patients were matched as a sensitivity analysis according to age, LVEF, EuroScoreII and primary/secondary MR; this resulted in two cohorts of n = 49 patients each, comparable regarding these parameters (Supplementary Table S1). Mean age in the matched cohorts was 71.7 years (95% CI: 69.3–74.1) in M-TEER vs. 70.0 years (95% CI: 67.9–72.1) in MIC-MVS (*p* = 0.30). Of note, in the matched M-TEER cohort, patients still showed coronary artery disease and previous PCI procedures (20.0% vs. 55.0%, *p* = 0.05) more frequently, had a lower GFR and TR was significantly more pronounced, being moderate or more in 59.2% vs. 22.4% (*p* < 0.01). Baseline characteristics of the matched cohorts are shown in Table 1.

**Figure 2:**
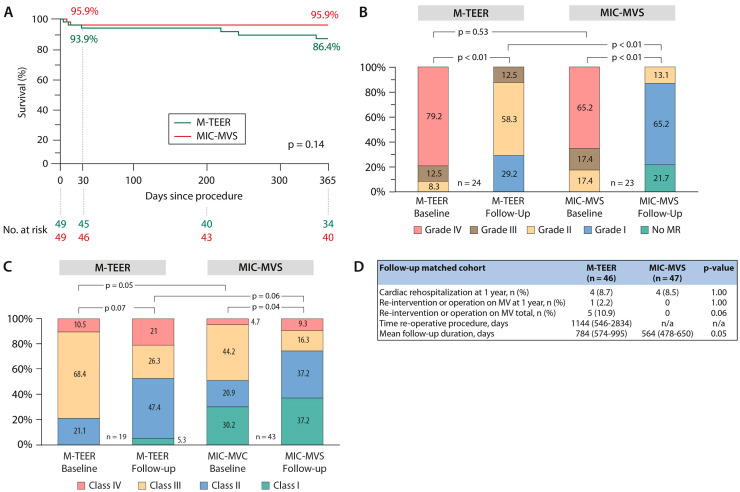
Study results of the matched cohort. (**A**) Kaplan–Meier curves of one-year survival in the mitral transcatheter edge-to-edge valve repair (M−TEER) and minimally invasive mitral valve surgery (MIC−MVS) groups. (**B**) Changes in the severity of MR grades at one year follow-up for both procedures as paired analyses. (**C**) Distribution of NYHA classes at baseline and one year as paired analyses. (**D**) Follow−up data. Legend: M−TEER = mitral transcatheter edge−to−edge repair valve repair; MIC-MVS = minimally invasive mitral valve surgery; NYHA = New York Heart Association; MR = mitral regurgitation; MV = mitral valve.

The sensitivity analysis by matching demonstrated a comparable survival rate between groups at 30 days (93.9% vs. 95.9%) and at 1 year (86.4% vs. 95.9%), *p* = 0.14 (Figure 2A).

In the matched cohort, a MitraClip device was used in M-TEER in 98.0% (48/49) and the mitral valve repair rate in MIC-MVS was 100%. In this matched cohort presenting with mainly primary MR, reduction to at least a moderate degree was achieved in 87.6% in M-TEER vs. 100% in MIC-MVS (differences in MR grade at discharge: *p* < 0.01), whereas the gradient was marginally higher after M-TEER (*p* = 0.04) (Table 2).

This analysis showed an improved grade of MR in both cohorts, which was more pronounced in MIC-MVS patients: MR was reduced to none or mild in 70.8% (34/48) in M-TEER and in 87.3% (41/47) in MIC-MVS patients (*p* < 0.01) at one-year follow-up (Figure 2B). MR reduction was sustained, as can be seen in Figure 2B.

There was significant improvement in NYHA class during follow-up in both cohorts (Figure 2C).

Mortality, stroke rate, cardiac rehospitalization and other postoperative severe adverse events were comparable between the groups (Table 2 and Figure 2D). In the matched cohorts, one patient in M-TEER needed ECMO therapy due to a STEMI with low-cardiac output and three patients needed ECMO therapy after MIC-MVS. The reasons were as follows: One was due to pulmonary edema caused by obstruction of the pulmonary vein by the left atrial suture line in a very comorbid patient with fatal outcome. One was due to hemodynamic instability based on underlying pulmonary arterial hypertension and residual MR after MIC-MVS with need for temporary ECMO therapy but ultimately full recovery. The third ECMO was necessary in a patient who, after an initially uneventful course, developed a sudden pericardial effusion on the normal ward with cardiopulmonary resuscitation and prolonged vasopressor therapy after therapeutic drainage with ultimately fatal outcome.

### 3.5. Patients with Degenerative Mitral Valve Disease

Table 3 shows data on patients with degenerative mitral valve disease within the overall and matched cohorts. This subanalysis shows significant differences int the overall cohort, e.g., patients with degenerative mitral valve disease were significant older in the M-TEER group, 80.3 years (95% CI: 79.3–81.2) vs. 60 years (95% CI: 57.2–62.8) (*p* < 0.01), and M-TEER patients had a higher EuroSCORE II, 5.4% (95% CI: 4.8–5.9) vs. 1.2% (95% CI: 1.0–1.3) (*p* < 0.0.1). MR reduction was still more effective in MIC-MVS patients as reduction to none or a mild degree was successful in 69.3% vs. 79.4% of patients in the overall (*p* < 0.01) and 62.5% vs. 80% in the matched cohort (*p* = 0.03). Re-intervention rate at one year was not significantly different, but was still higher after M-TEER during further follow-up.

**Figure 3:**
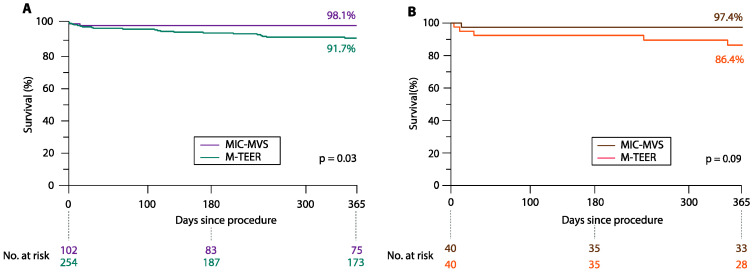
Survival rates of patients with degenerative mitral valve disease—subanalysis. (**A**) Kaplan–Meier curves of one-year survival in patients with degenerative mitral valve disease after mitral transcatheter edge-to-edge valve repair (M-TEER) and minimally invasive mitral valve surgery (MIC-MVS) in the overall cohort. (**B**) Kaplan–Meier curves of one-year survival in patients with degenerative mitral valve disease after mitral transcatheter edge-to-edge valve repair (M-TEER) and minimally invasive mitral valve surgery (MIC-MVS) in the matched cohort. Legend: M-TEER = mitral transcatheter edge-to-edge repair valve repair; MIC-MVS = minimally invasive mitral valve surgery.

Kaplan–Meier survival analyses showed a 91.7% survival rate at one year in M-TEER and 98.1% in MIC-MVS (*p* = 0.03) in the overall cohort and 86.4% vs. 97.4% in the matched cohort (*p* = 0.09) (Figure 3A,B).

## 4. Discussion

This analysis is the largest comparison between interventional mitral TEER and surgical minimally invasive procedures in patients with mitral regurgitation to date. In this contemporary setting of more than 850 subjects, M-TEER had lower one-year survival than MIC-MVS but differences disappeared after matching. Substantial MR reduction was achieved in both groups at discharge and 1 year follow-up, but there was a significantly greater reduction in MR in patients after surgical treatment. These findings were statistically confirmed by a sensitivity analysis.

Although intermediate surgical risk (or moderate risk) has been well defined for aortic stenosis, such a population is not well defined for mitral valve repair surgery, where operative mortality rates are usually low and a frequently used minimally invasive access results in faster post-operative recovery. Consequently, surgical risk stratifications in the guidelines differ for surgical mitral valve repair and surgical aortic valve replacement [6].

M-TEER is the most widely used transcatheter technique for patients with MR, and the effectiveness of TEER procedures in reducing MR and improving clinical prognosis has been proven [6,7].

In the past decade, minimally invasive surgical techniques have overtaken conventional approaches to mitral valve surgery and have improved patient outcomes. These procedures allow the surgeon to preserve mitral valve anatomy and restore valve function, usually with no residual MR, primarily in degenerative mitral valve disease. In the case of MR recurrence, or if other cardiac operations would become necessary, there are no issues related to sternal re-entry with much fewer adhesions since the ventral side of the pericardium is still intact and a surgical re-repair might still be feasible. Also, minimally invasive mitral procedures might be combined with other procedures, such as atrial ablation, left atrial appendage closure or other valve surgeries.

However, many MR patients are at high surgical risk and therefore catheter-based interventions for mitral valve disease are a routinely performed alternative. Currently, in elderly intermediate-risk patients there is a rising discussion about the adequate therapeutical approach to MR. In the current study, we aimed to compare the two MR therapy techniques. The analysis of overall cohorts shows that currently M-TEER and MIC-MVS treat entirely different patient populations with very different risk profiles. This reflects the practice of heart team-based decision making in a large heart valve center. Given the confounders affecting treatment allocation, we applied a matching to balance the differences in baseline characteristics between interventional and surgical patients. Even after matching, some differences remained. The matched study groups consisted of patients around 70 years of age. Despite some differences in the matched cohorts, particularly in patients’ history of coronary artery disease and intervention, our analysis showed that patients who underwent M-TEER or MIC-MVS appeared to have a comparable survival rate at 30 days and 1 year.

The present findings suggest that MIC-MVS leads to more bleeding complications and longer hospital stays than M-TEER. M-TEER was safe, with a very low incidence of peri-operative adverse events.

MR reduction was significantly less efficient in M-TEER, but this did not lead to a higher rate of rehospitalization at one year. In some patients with severe heart failure, complete reduction of MR may increase afterload. These patients should be treated carefully.

After M-TEER, the re-intervention/operation rate for MV disease remains higher in the long term, whether this translates to impaired survival or more pronounced clinical symptoms in the long-term is yet unclear. Generally, it has to be determined in which patients MR reduction confers to a prognostic benefit, as was the case in two landmark trials (COAPT and MITRA-FR) [13,14]. In contrast to these studies, our analysis of matched patients consisted mainly of patients with primary mitral valve disease. In a large French survey, it was shown that a relatively large number of patients with severe MR were referred to surgery, indicating an unmet need for severe MR treatment: a significant increase in mitral TEER procedures was observed in France, and compared to isolated surgical MR treatment [15] TEER patients showed a lower cardiovascular mortality than mitral surgery patients at a long-term follow-up. There were no differences in all-cause mortality between the TEER and the surgical group. MR reduction was not reported. These results are in line with our results. But Deharo et al. [15] depicted a comparative study on TEER vs. surgery including sternotomy approaches, but none on solely TEER vs. MIC-MVS, which is the more evolved procedure. It is clearly less traumatic with evidence of superior postoperative recovery. Unmatched data were not presented.

In our study, we were unable to find short- or mid-term differences in survival between the two techniques in matched cohorts and, therefore, we are convinced that larger and prospective studies should be performed to further evaluate our findings and to assist heart teams in the decision-making process in patients with mitral valve disease. Results of our analysis, especially baseline characteristics after matching, may help define intermediate-risk patient cohorts for further randomized clinical trials. Of note, these trials should not consider reduction in MR to a moderate degree as success, since M-TEER procedures should be held to the same standards as surgical procedures. Furthermore, future randomized controlled studies should focus on comparison of M-TEER with minimally invasive techniques, as they have become standard in heart valve centers across Europe.

### Limitation

Limitations of our study are apparent through its retrospective design with inherent consequences such as bias and possible differences in patient characteristics despite matching. Irrespective of the different therapies applied, a selection bias is due to the fact that, in general, M-TEER was performed in sicker patients overall. Furthermore, sample size of our study was limited and conclusions should be drawn carefully and are mainly seen as hypothesis-generating. There was no independent corelab assessment of echocardiographic findings. We did not analyze data concerning left ventricular remodeling. There was no data collection according to Mitral Valve Academic Research Consortium criteria [16].

Another notable limitation is that these data encompass some of the early experiences with the M-TEER devices and thus do not fully reflect the current experience level of the individual M-TEER operators.

Nonetheless, this is the first study with a direct comparison of M-TEER versus MIC-MVS in patients with MR.

## 5. Conclusions

To our knowledge, this is the first direct comparative analysis of outcomes after M-TEER versus MIC-MVS. Although MIC-MVS leads to more effective reduction in MR, we observed comparable survival at 30 days and one year. M-TEER patients may need more frequent re-intervention or operation on the MV in the long-term, but this did not result in a higher rate of rehospitalization for heart failure. Notably, this was a selection of elderly patients at presumed intermediate risk. Proper patient selection for treatment of MR seems to be key in achieving improved outcomes. The efficacy of M-TEER in terms of MR reduction and the role of catheter-based valve replacement in these patients needs further assessment. Our analysis lays the basis for further prospective comparative studies of M-TEER vs. MIC-MVS, since it helps define possible intermediate risk patient populations for a comparison of these techniques.

## Figures and Tables

**Table 1:** Baseline Parameters of the M−TEER and MIC−TVS Patients in the overall and matched Cohort.

Baseline Parameters	Before Matching			After Matching		
	M-TEER (n = 723)	MIC-MVS (n = 123)	*p*-Value	M-TEER (n = 49)	MIC-MVS (n = 49)	*p*-Value
Age, years	78.3 (77.7–78.8)	61.5 (59.0–63.9)	<0.01	71.7 (69.3–74.1)	70.0 (67.9–72.1)	0.30
Sex (female), n (%)	305 (42.2)	52 (42.3)	0.98	21 (42.9)	25 (51.0)	0.42
BMI, kg/m^2^	26.1 (25.6–26.6)	26.3 (25.5–27.2)	0.59	25.5 (23.9–27.1)	26.7 (25.3–28.1)	0.27
Logistic EuroSCORE II, %	5.5 (5.2–5.9)	1.3 (1.1–1.5)	<0.01	2.3 (1.9–2.7)	1.8 (1.4–2.2)	0.08
LVEF, %	48.4 (47.3–49.6)	60.4 (58.8–62.0)	<0.01	60.0 (57.7–62.2)	60.5 (58.4–62.4)	0.73
Glomerular filtration rate, mL/min	45.9 (44.3–47.5)	93.5 (86.8–100.1)	<0.01	60.6 (52.7–68.6)	76.3 (67.4–85.3)	0.01
Previous Cardiac Surgery, n (%)	183 (25.3)	2 (1.6)	<0.01	4 (8.2)	0	0.04
COPD, n (%)	132 (18.2)	11 (8.9)	<0.01	12 (24.5)	6 (12.2)	0.12
Previous Stroke/TIA, n (%)	70 (9.7)	7 (5.7)	<0.01	4 (8.2)	2 (4.1)	0.34
Coronary artery disease, n (%)	403 (55.7)	16 (13.0)	<0.01	7 (14.3)	4 (8.2)	<0.01
Previous PCI, n (%)	284 (39.3)	4 (3.3)	<0.01	17 (34.7)	3 (6.1)	<0.01
Permanent Pacemaker/internal Defibrillator present, n (%)	251 (34.7)	6 (4.9)	<0.01	6 (12.2)	1 (2.0)	0.05
Creatinine, mg/dL	1.5 (1.4–1.6)	0.9 (0.9–1.0)	<0.01	1.2 (1.0–1.5)	1.0 (0.9–1.1)	0.07
History of atrial fibrillation/atrial flutter, n (%)	546 (75.5)	40 (32.5)	<0.01	34 (69.4)	20 (40.8)	<0.01
Systolic PAP, mmHg	42.2 (41.1–43.4)	45.6 (41.9–49.4)	0.02	42.9 (37.7–48.2)	45.6 (41.1–50.1)	0.47
TAPSE, mm	18.4 (17.9–18.8)	23.7 (22.3–25.2)	<0.01	20.2 (18.6–21.8)	25.4 (23.7–27.1)	<0.01
Vena contracta MV, mm	0.7 (0.6–0.7)	0.7 (0.6–0.7)	1.00	0.7 (0.7–0.8)	0.7 (0.6–0.8)	0.95
LVEDD, mm	52.7 (51.8–53.7)	51.1 (49.3–53.0)	0.12	51.0 (48.7–53.3)	50.4 (47.7–53.2)	0.37
TR Grade, n (%) None Mild Moderate Severe Massive Torrential			<0.01			<0.01
	9/709 (1.3) 200/709 (28.2) 259/709 (36.5) 171/709 (24.1) 58/709 (8.2) 12/709 (1.7)	36 (29.3) 56 (45.5) 15 (12.2) 13 (10.6) 3 (2.4) 0		1 (2.0) 19 (38.8) 19 (38.8) 8 (16.3) 2 (4.1) -	9 (18.4) 29 (59.1) 9 (18.4) 2 (4.1) 0 -	<0.01

Values are either n (%) or continuous values, which are given as mean values with 95% lower and upper confidence intervals. Abbreviations: M-TEER = mitral transcatheter edge-to-edge repair valve repair; MIC-MVS = minimally invasive mitral valve surgery; BMI = body mass index; LVEF = left ventricular ejection fraction; COPD = chronic obstructive pulmonary disease; TIA = transitory ischemic attack; PCI = percutaneous coronary intervention; PAP = pulmonary artery pressure; TAPSE = tricuspid annular plane systolic excursion; LVEDD = left ventricular end-diastolic diameter; MR = mitral regurgitation; TR = tricuspid regurgitation.

**Table 2:** Procedural and follow-up Data of the M−TEER and MIC−TVS Patients in the overall and matched Cohort.

	Before Matching			After Matching		
Procedural Data and 30-day Results	M-TEER (n = 723)	MIC-MVS (n = 123)	*p*-Value	M-TEER (n = 49)	MIC-MVS (n = 49)	*p*-Value
Procedure time, minutes	78.3 (77.7–78.8)	61.5 (59.0–63.9)	<0.01	72.8 (60.5–85.1)	266.8 (247.4–286.3)	<0.01
Time on ECC, minutes Cross Clamp time, minutes	n/a	192.5 (182–202)		n/a		
Time on ECC, minutes Cross Clamp time, minutes	n/a	127.1 (120–134)		n/a		
Mitral valve repair (surgical), n (%) Mitral valve replacement (surgical), n (%) Method of TEER, n (%) MitraClip Pascal Number of clips placed, n (%): 1 Clip 2 Clips 3 Clips	n/a n/a 691 (95.6) 32 (4.4) 380 (52.6) 299 (41.3) 44 (6.1)			48 (98.0) 1 (2.0) 48 (98.0) 1 (2.0) 24 (48.9) 21 (42.9) 4 (8.2)		n/a
Concomitant procedures, n (%) Pacemaker lead extraction Rhythm ablation therapy LAA closure PFO/ASD closure Tricuspid valve surgery	n/a	0 21 (17.1) 18 (14.6) 16 (13.0) 17 (13.8)		n/a	0 9 (18.4) 5 (10.2) 6 (12.2) 0	0.27
Conversion to sternotomy, n (%)	5 (0.7)	6 (4.9)	<0.01	0	3 (6.1)	0.34
30-day mortality, n (%)	26 (3.6)	3 (2.4)	0.78	3 (6.1)	2 (4.1)	1.00
TIA, n (%) Stroke, n (%)	0 6 (0.8)	0 1 (0.8)	1.00 1.00	0 0	0 1 (2.0)	1.00 1.00
Major bleeding (BARC 3 or 4), n (%)	24 (3.3)	23 (18.7)	<0.01	2 (4.1)	8 (16.3)	0.09
LCOS with, n (%) VA ECMO left heart Impella right heart Impella	2 (0.3) 1 (0.1) 0	5 (4.1) 1 (0.8) 0	<0.01 0.27 1.00	1(2.0) 0 0	3 (6.1) 0 0	0.62
Renal failure requiring dialysis, n (%)	4 (0.5)	7 (5.7)	<0.01	1 (2.0)	4 (8.2)	0.36
Hospital stay, days	7.0 (6.6–7.5)	18.1 (14.9–21.3)	<0.01	5.7 (4.6–6.9)	23.5 (16.4–30.5)	<0.01
MR severity at discharge, n (%) None Mild Moderate Moderate-Severe Severe	46/696 (6.6) 479/696 (68.8) 131/696 (18.8) 28/696 (4.0) 12/696 (1.7)	38/107 (35.5) 61/107 (57.0) 6/107 (5.6) 2/107 (1.9) 0	<0.01	1/48 (2.1) 33/48 (68.8) 8/48 (16.7) 4/48 (8.3) 2/48 (4.1)	21/47 (44.7) 20/47 (42.5) 6/47 (12.8) 0 0	<0.01
Mitral mean gradient at discharge, mmHg	3.8 (3.6–3.9)	3.6 (3.3–3.9)	0.32	4.1 (3.6–4.6)	3.4 (3.0–3.8)	0.04

Values are either n (%) or continuous values, which are given as mean values with 95% lower and upper confidence intervals. Abbreviations: M-TEER = mitral transcatheter edge-to-edge repair valve repair; MIC-MVS = minimally invasive mitral valve surgery; MR = mitral regurgitation; ECC = extracorporeal circulation; TEER = transcatheter edge-to-edge repair; LAA = left atrial appendage; PFO = patent foramen ovale; ASD = atrial septal defect; BARC = Bleeding Academic Research Consortium; LCOS = low cardiac output syndrome; VA ECMO = veno-arterial extracorporeal membrane oxygenation.

**Table 3:** Subanalysis of Patients with degenerative Mitral Valve Disease after M−TEER and MIC−TVS in the overall and matched Cohort.

Parameters	Before Matching			After Matching		
	M-TEER (n = 254)	MIC-MVS (n = 102)	*p*-Value	M-TEER (n = 40)	MIC-MVS (n = 40)	*p*-Value
Age, years	80.3 (79.3–81.2)	60.0 (57.2–62.8)	**<0.01**	72.0 (69.1–74.9)	70.1 (67.6–72.7)	0.33
Logistic EuroSCOREII, %	5.4 (4.8–5.9)	1.2 (1.0–1.3)	**<0.01**	2.1 (1.8–2.4)	1.6 (1.3–1.9)	**<0.01**
30-day mortality, n (%)	8 (3.1)	2 (1.9)	0.73	3 (7.5)	1 (2.5)	0.62
MR severity at discharge, n (%) None Mild Moderate Moderate-Severe Severe	28/244 (11.5) 148/244 (60.6) 51/244 (20.9) 11/244 (4.5) 6/244 (2.5)	30/89 (33.7) 51/89 (57.3) 8/89 (9.0) 0 0	**<0.01**	5/38 (13.2) 20/38 (52.5) 9/38 (23.7) 2/38 (5.3) 2/38 (5.3)	16/38 (42.1) 16/38 (42.1) 6/38 (15.8) 0 0	**0.03**
Mitral mean gradient at discharge, mmHg	4.0 (3.8–4.3)	3.5 (3.2–3.8)	**0.01**	4.1 (3.5–4.7)	3.2 (2.8–3.6)	**0.02**
1-year mortality, n (%)	21 (8.3)	2 (1.9)	**0.03**	5 (12.5)	1 (2.5)	0.20
Cardiac rehospitalization at 1 year, n (%)	20 (7.9)	4 (3.9)	0.24	2 (5.0)	2 (5.0)	1.00
Re-intervention or operation on MV at 1 year, n (%)	7 (2.8)	0	0.20	1 (2.5)	0	1.00
Re-intervention or operation on MV total, n (%)	19 (7.5)	0	**<0.01**	5 (12.5)	0	**0.06**
Mean follow-up duration, days	728 (639–817)	528 (463–593)	**<0.01**	846 (596–1097)	580 (483–678)	**0.05**

Values are either n (%) or continuous values, which are given as mean values with 95% lower and upper confidence intervals. Significant values are given in bold letters. Abbreviations: M-TEER = mitral transcatheter edge-to-edge repair valve repair; MIC-MVS = minimally invasive mitral valve surgery; MR = mitral regurgitation; MV = mitral valve.

## Data Availability

The data presented in this study are available on request from the corresponding author. The data are not publicly available due to confidentiality and German data protection laws.

## Supplementary Materials

The following supporting information can be downloaded at: https://www.mdpi.com/article/10.3390/jcm13051372/s1, Figure S1: Violin plots showing covariate balance; Table S1: Matching Parameters.
