# Supplementary material for: Transcatheter Edge-to-Edge Mitral Valve Repair versus Minimally Invasive Mitral Valve Surgery: An Observational Study
Source: J Clin Med. 2024 Feb 28;13(5):1372. doi: 10.3390/jcm13051372 (PMC10932335; doi:10.3390/jcm13051372)
Supplement: Supplementary file 1 [file jcm-13-01372-s001.zip › jcm-2829916-supplementary.pdf]

Supplemental Table S1. Matching parameters.

Variable	Before Matching M-TEER (n = 723)	Before Matching MIC-MVS (n = 123)	p-value	After Matching M-TEER (n = 49)	After Matching MIC-MVS (n = 49)	p-value
Age, years	78.3 (77.7-78.8)	61.5 (59.0-63.9)	<0.01	71.7 (69.3-74.1)	70.0 (67.9-72.1)	0.30
LVEF, %	48.4 (47.3-49.6)	60.4 (58.8-62.0)	<0.01	60.0 (57.7-62.2)	60.5 (58.4-62.4)	0.73
EuroSCORE II, %	5.5 (5.2-5.9)	1.3 (1.1-1.5)	<0.01	2.3 (1.9-2.7)	1.8 (1.4-2.2)	0.08
MR etiology, n (%)						1.00
Primary MR	254 (35.1)	102 (82.9)		40 (81.6)	40 (81.6)	
Secondary MR	428 (49.2)	21 (17.1)		9 (18.4)	9 (18.4)	
Other (i.e. previous Clip), n (%)	41 (5.7)	0		–	–	

Abbreviations: M-TEER = transcatheter edge-to-edge repair valve repair; MIC-MVS = minimally invasive mitral valve surgery; LVEF = left ventricular ejection fraction; SMD = standard mean difference; M-TEER = transcatheter edge-to-edge repair valve repair; MR = mitral regurgitation.

Supplemental Figure S1. Violin plots showing covariate balance.

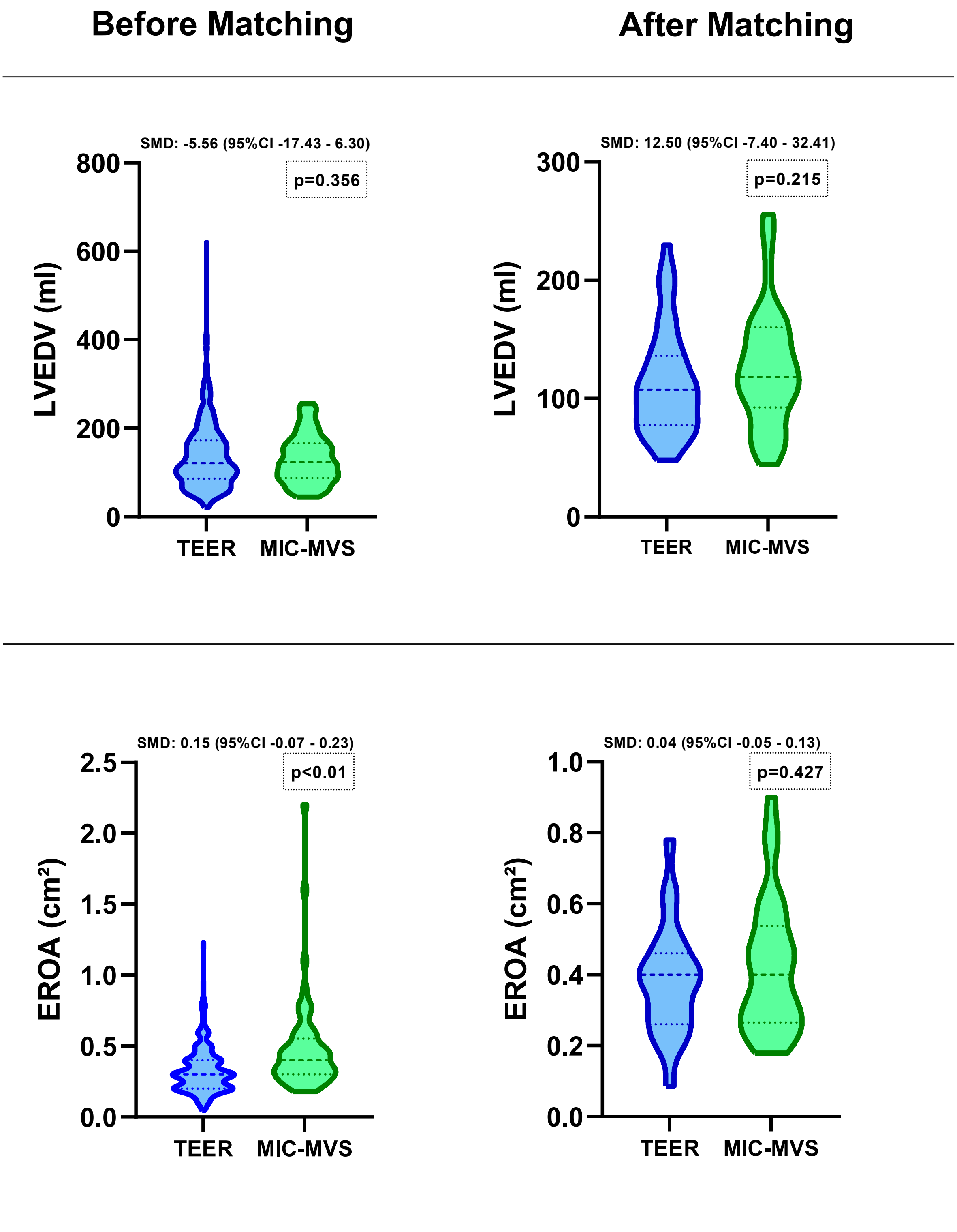

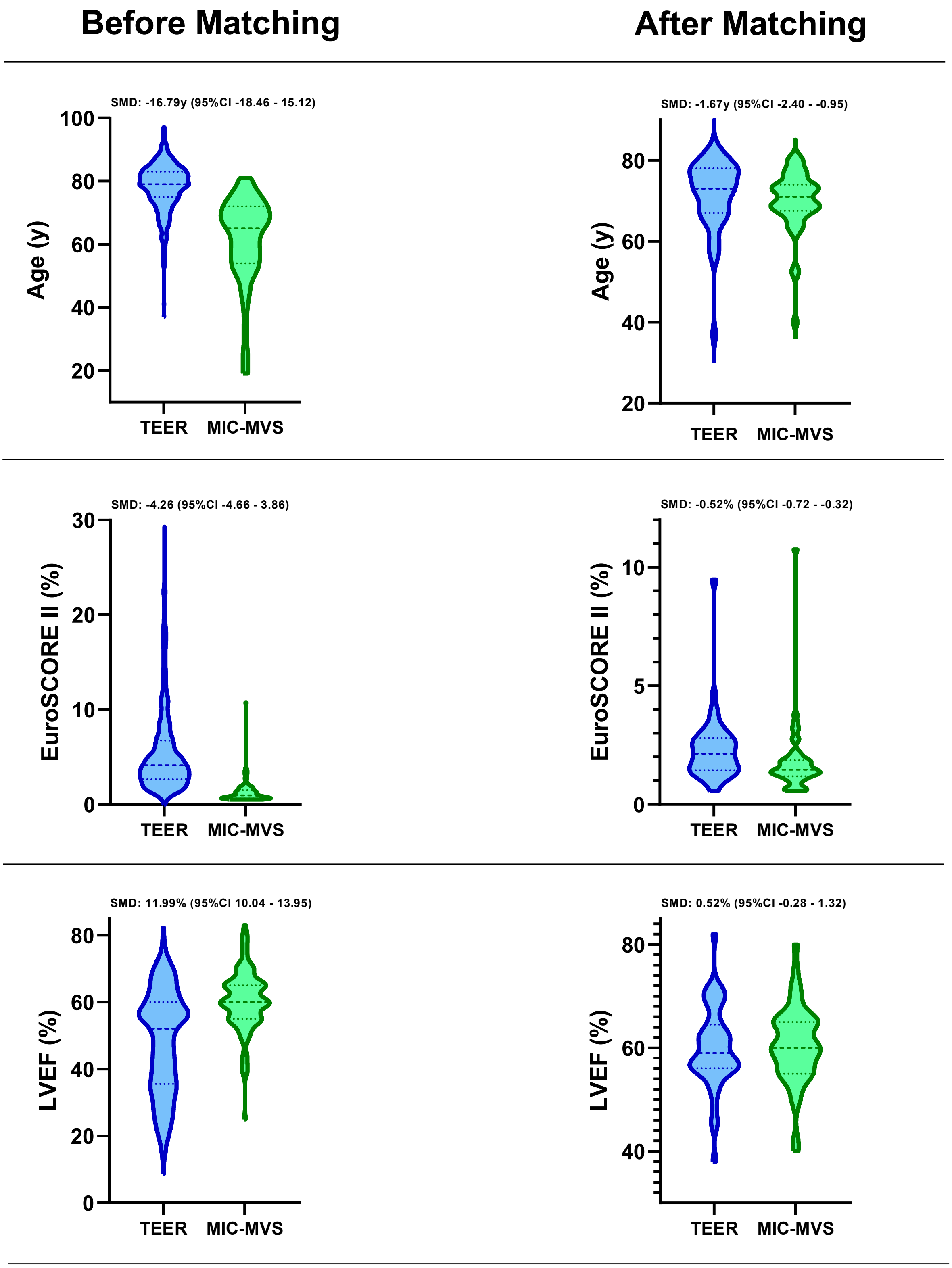

Abbreviations: EROA = effective regurgitation orifice area; MIC-MVS = minimally invasive mitral valve surgery; LVEDV = left ventricular end-diastolic volume; LVEF = left ventricular ejection fraction; SMD = standard mean difference; TEER = transcatheter edge-to-edge repair valve repair.
